# Durable Clinical Responses and Long-Term Follow-Up of Stage III–IV Non-Small-Cell Lung Cancer (NSCLC) Patients Treated With IDO Peptide Vaccine in a Phase I Study—A Brief Research Report

**DOI:** 10.3389/fimmu.2018.02145

**Published:** 2018-09-19

**Authors:** Julie Westerlin Kjeldsen, Trine Zeeberg Iversen, Lotte Engell-Noerregaard, Anders Mellemgaard, Mads Hald Andersen, Inge Marie Svane

**Affiliations:** ^1^Department of Oncology, Herlev Hospital, University of Copenhagen, Herlev, Denmark; ^2^Department of Hematology, Center for Cancer Immune Therapy, Herlev Hospital, University of Copenhagen, Herlev, Denmark

**Keywords:** cancer, immunotherapy, NSCLC, IDO, peptide vaccine

## Abstract

**Background:** Long-term follow-up on a clinical trial of 15 stage III-IV NSCLC patients treated with an Indoleamine 2,3-Dioxygenase (IDO) peptide vaccine (NCT01219348).

**Methods:** Fifteen HLA-A2-positive patients with stable stage III-IV NSCLC after standard chemotherapy were treated with subcutaneous vaccinations (100 μg IDO5 peptide, sequence ALLEIASCL, formulated in 900 μl Montanide) biweekly for 2.5 months and thereafter monthly until progression or up to 5 years. Here we report long-term clinical follow-up, toxicity and immunity.

**Results:** Three of 15 patients are still alive corresponding to a 6-year overall survival of 20 %. Two patients continued monthly vaccinations for 5 years (56 vaccines). One of the two patients developed a partial response (PR) of target lesions in the liver 15 months after the first vaccine and has remained in PR ever since. The other patient had a solitary distant metastasis in a lymph node in retroperitoneum at baseline which normalized during treatment. All following evaluation scans during the treatment have been tumor free. The vaccine was well tolerated for all 5 years with no long-term toxicities registered. The third long-term surviving patient discontinued vaccinations after 11 months due to disease progression. Flow cytometry analyses of PBMCs from the two long-term responders demonstrated stable CD8+ and CD4+ T-cell populations during treatment. In addition, presence of IDO-specific T-cells was detected by IFN-γ Elispot in both patients at several time points during treatment.

**Conclusion:** IDO peptide vaccination was well tolerated for administration up to 5years. Two of 15 patients are long-term responders with ongoing clinical response 6 years after 1st vaccination.

## Introduction

Lung cancer is the leading cause of cancer death in both men and women worldwide, with non-small-cell lung cancer (NSCLC) accounting for 85–90% (1). At the time of diagnosis most patients have stage III–IV inoperable disease with a poor prognosis and a 5-year overall survival of <5%.

Previously, first-line standard treatment for the majority of patients with metastatic NSCLC, when no targetable alteration is revealed, was platinum-based chemotherapy, but only 15–30% of the patients responded (2).

Cancer immunotherapy, a treatment that boosts the body's natural defense to fight cancer has greatly evolved the last decade, and is now the standard of choice in many solid tumors. Nivolumab and Pembrolizumab, both PD-1 blocking antibodies and Atezolizumab a PD-L1 blocking antibody are approved by FDA and EMA for second line treatment for NSCLC and Pembrolizumab as first line treatment for patients with tumors expressing PD-L1 (3–5). All three antibodies work by relieving the suppression of the anti-tumor immunity, thereby boosting the immune system to kill cancer cells. Multiple immune regulatory targets are being investigated these days, among others indoleamine 2,3- dioxygenase (IDO).

IDO is an intracellular enzyme that catalyzes the rate-limiting step in degradation of Tryptophan (T) leading to local depletion and an increase in Kynurenine (K) metabolites (6). An upregulation of IDO in tumor cells leads to depletion of T which suppresses T-cell function and survival (7). Because T and K concentration can be measured from patients' serum, IDO activity can be monitored by computing K/T ratio (8). Consequently, cancer patients, including lung cancer, exhibit higher K/T ratios compared to healthy donors suggesting elevated IDO activity in cancer patients, thus proposing IDO as a valuable target in cancer.

IDO-specific T-cells have been shown to influence adaptive immune reactions in both cancer patients and healthy donors. Further, we have shown that these IDO-specific T-cells are cytotoxic effector cells capable of recognizing and killing both cancer cells and immunosuppressive dendritic cells *in vitro*. These findings justified clinical testing of an IDO derived peptide vaccine with the aim of boosting the IDO specific cytotoxic T-cells (9). A phase I vaccination study was performed at our institution from 2010 to 2012 including 15 HLA-A2+ stage III/IV NSCLC patients, demonstrating significant improved overall survival when compared with the group of excluded patients because of HLA-A2 negativity (10). Here, we present the long-term clinical and immunological outcomes of the treatment.

## Materials and methods

### Patients

Fifteen HLA-A2 positive patients with biopsy verified stage III–IV NSCLC in stable disease after standard chemotherapy were treated with subcutaneous vaccinations (100 μg IDO5 peptide, sequence ALLEIASCL, formulated in 900 μl of the adjuvant Montanide) (11). This study was carried out in accordance with the recommendations of GCP with written informed consent from all subjects. All subjects gave written informed consent in accordance with the Declaration of Helsinki. The protocol was approved by the National Board of Health and the local Ethics Committee at the Capital Region of Denmark. The initial study (NCT01219348) results have previously been reported (10). Patients were enrolled from June 2010 to May 2012 and treated every second week for 2.5 months and thereafter monthly until progression or up to 5 years. Two of the 15 patients have completed 5 years of vaccination, enabling evaluation of potential long-term toxicity according to CTCAE version 4.0. Furthermore, long-term clinical benefit was evaluated by CT or PET-CT scans according to Response Evaluation Criteria in Solid Tumors 1.1 (RECIST 1.1) at baseline and every third month for a completion of 5 years follow-up.

### Patient material

Peripheral blood mononuclear cells (PBMC) were obtained from peripheral blood by Lymphoprep technique by gradient centrifugation every third month during vaccination from the two long-term responders. Isolated cells were frozen immediately with 90% humanized AB-serum and 10% dimethyl sulfoxide and stored at −180°C.

### Elispot

To assess whether IDO vaccination resulted in measurable T-cell responses in the two long-term patients, we performed indirect IFN- ELISPOT as previously described. Briefly, PBMCs were stimulated once in *ex vivo* medium +5% HS, 120 U/L interleukin-2 and 15 umol/L IDO5 peptide prior to analysis to extend the sensitivity of the assay. After 7 days in culture, cells were counted and analyzed in IFN-y ELISPOT. Nitrocellular bottomed 96-well plates (MultiScreen MAIP N45; Millipore) were coated with IFN-y capture mAb (Mabtech) overnight. Wells were washed, blocked by *X-vivo* medium and the effector cells were added in duplicates at different concentrations with or without 5 umol/L of the IDO5 peptide. Plates were incubated overnight and medium was discharged and wells washed prior to addition of biotinylated secondary Ab (Mabtech). Plates were incubated at room temperature (RT) for 2 h, washed and avidin-enzyme conjugate was added to each well. Plates were incubated at RT for 1 h and the enzyme substrate NBT/BCIP (Invitrogen Life Technologies) was added to each well and incubated at RT for 5–10 min. Upon the emergence of dark purple spots, the reaction was terminated by washing with tap water. The spots were counted using the ImmunoSpot Series 2.0 Analyzer (CTL Analyzers).

### Flow cytometry

PBMC samples were thawed in 37°C RPMI medium 1640 + GlutaMAX (Life Technologies) and thereafter washed in RPMI and stained in PBS containing 0.5% bovine serum albumin. For phenotyping of CD3+ T-cells, the following antibodies were used: CD45RA-FITC, CD62L-PE, CCR7-PE-CY7, CD3-APC, CD8-BV421, CD4-HV510 (BD Biosciences), CD27-PerCP (Nordic Biosite). Natural Killer cells, B-cells, and γ/δ cells were stained with the following antibodies: CD16-FITC, CD56-PE, CD19-PE-CY7, CD3-APC (BD Biosciences), and γ/δ -BV421 (Nordic Biosite). Myeloid derived suppressor cells were stained with: CD33-FITC, HLA-DR-PerCP, lineage = CD3-, CD19-, and CD56-PE-Cy7, CD11b-APC (BD Biosciences), CD14-BV421 (Nordic Biosite). Regulatory T-cells were stained with CD45RA-FITC, CCR4-PerCP-Cy5.5, CD127-PE-Cy7, CD4-APC, CD25-BV421 (BD Biosciences), FoxP3-PE (eBiosciences). Dead cell marker APC-Cy7 near IR (Invitrogen) fluorescent reactive dye was used to exclude dead cells. For intracellular staining of transcription factor FoxP3, we used Transcription Factor Staining Buffer set (eBioscience) according to guidelines issued by the manufacturer.

## Results

### Long-term clinical follow-up

**Figure 1:**
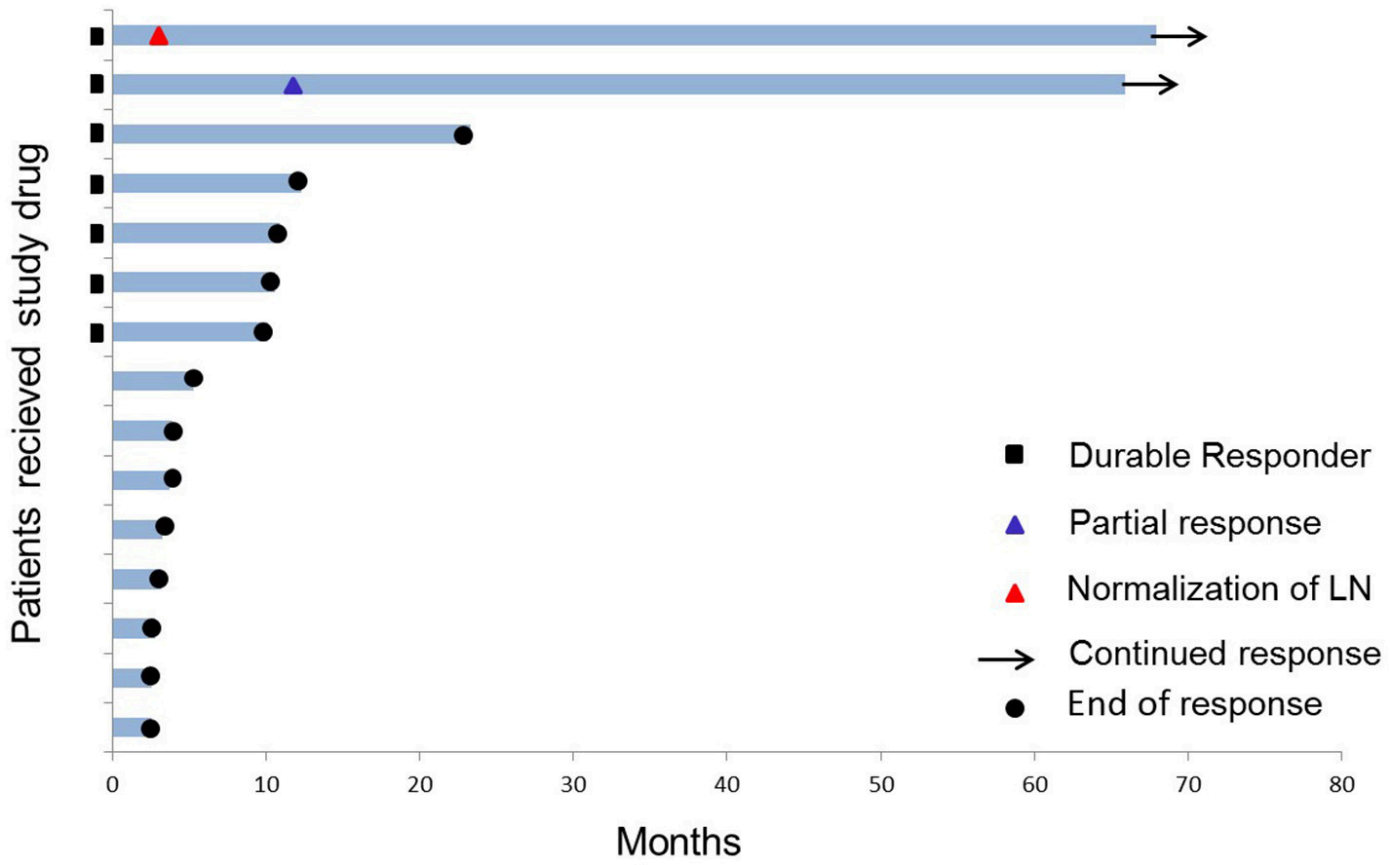
Swimmer plot of the 15 stage III–IV HLA-A2+ NSCLC patients who received study drug (IDO peptide vaccine). Two of 15 patients are long-term responders (as of 6 years after the 1st vaccine). Durable response is defined as >8.5 months clinical treatment benefit.

Three of the 15 patients are still alive (as of May 2018) corresponding to a 6-year overall survival of 20% (Figure 1). One patient was excluded from the trial due to progression after 11 months; the two other patients continued to be on monthly vaccination for 5 years with no other anti-cancer therapy given. They each received a total of 56 vaccines. Both patients had IDO expressing tumors (30–50%) by immunohistochemistry (10).

One of the two long-term responders (#18) was diagnosed with stage IV adenocarcinoma in 2009 (localized in lung and liver) and was initially treated with 1st line Carboplatin and Pemetrexed, 2nd line Erlotinib followed by 3rd line Docetaxel before inclusion in the trial in 2012. The patient achieved a partial response (PR) of target lesions in the liver 15 months after the first vaccine was administered and has been in ongoing stable PR for 6 years.

The other long-term responder (#17) was diagnosed with stage III adenocarcinoma in 2009; initially treated with an upper right lobectomy and subsequently 1. line Cisplatin and Vinorelbine. Further dissemination lead to a left adrenalectomy in 2010 due to a metastasis, followed 1 year later by 2. line Cisplatin and Pemetrexed for retroperitoneal lymph node recurrence before inclusion in the IDO vaccination trial in 2012. The patient had a solitary metastasis in a retroperitoneal gland (1.3 cm) at baseline which was normalized at 2nd evaluation during IDO vaccination. Absence of recurrent disease have been confirmed by CT ongoing for 6 years.

The third long-term survivor had stage IV disease and was treated with 4 lines of therapy before trial inclusion. The patient progressed after 11 months on IDO vaccination (14 vaccines administered) and was referred to standard of care where additional four lines of therapy have been given.

### Long-term toxicity

The vaccine was well-tolerated in both long-term responders receiving the vaccine for 5 years and no CTCAE grade 3–4 adverse events were observed. Both patients are in good performance status (PS 0) and only experienced grade 1 or 2 local reactions at the injection site; i.e., redness, itching, and subcutaneous granuloma. All three local reactions are known AEs to the adjuvant Montanide.

### Long-term immunity

**Figure 2:**
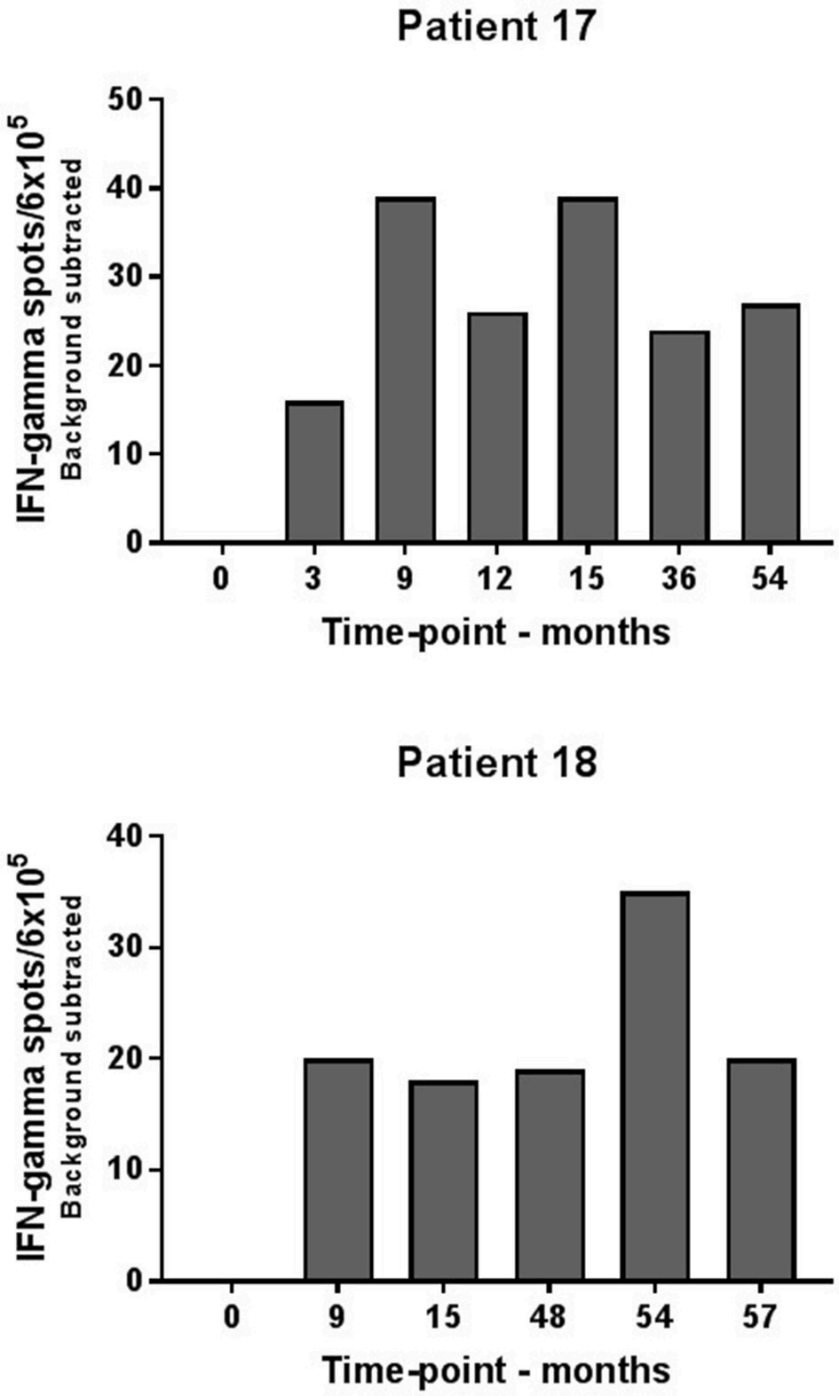
Elispot analysis of IDO-specific T-cells in consecutive blood samples from long-term responders. IDO specific T-cells were demonstrated in patient #17 and #18 at several time points during IDO vaccination course.

Consecutive ELISPOT analyses for evaluation of peripheral blood immune reactivity to the IDO peptide were established for the two long-term responders during their 5 years of treatment. Immune-monitoring demonstrated detectable vaccination-induced IDO specific T-cell responses at several time-points during vaccination on the two patients as opposed to baseline samples (Figure 2).

**Figure 3:**
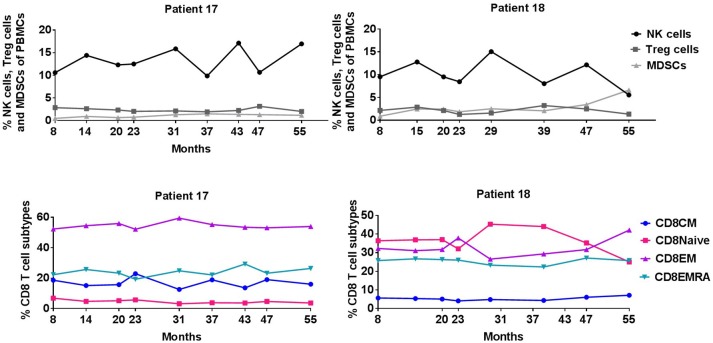
Percentage of NK cells, Treg cells, MDSCs, and CD8+ T cell subpopulations during IDO vaccination course.

Consecutive flow cytometry analyses of PBMCs during continuing vaccination (available from 8 to 56 months) were also performed on the two long-term responders (Figure 3). Peripheral blood percentages of CD8+ and CD4+ T-cells did not change significantly during vaccination as well as subpopulations of naïve, effector memory (EM), central memory and EMRA T-cells. Additional FACS analyses of natural killer (NK) cells, CD4+ regulatory T cells (Tregs), and myeloid derived suppressor cells (MDSCs) were also stable during vaccination for 5 years.

## Discussion

As published in 2013, vaccination in a phase I trial with an epitope derived from IDO in 15 patients with disease stabilization after standard chemotherapy demonstrated long-lasting PR+SD of at least 8.5 months in 47% of the patients (10). Historically, median PFS in patients with stage IV NSCLC treated with at least one line of chemotherapy is ~6–7 months (12). This long-term follow-up 6 years after IDO vaccine initiation shows a 20% 6-year overall survival as compared to historical data with a 5-year OS <5%. The improved OS obviously needs confirmation in a larger randomized clinical study. Still, two of 15 patients have ongoing clinical response 6 years after vaccination initiation and have not received additional anti-neoplastic treatment following the vaccination period.

Importantly, the two patients with ongoing clinical response have received 56 vaccines in total over 5 years, with only local and manageable side effects and no grade 3–4 toxicity reassuring the vaccine to be safe for administration for a long period.

Many vaccine trials in NSCLC have shown a vaccination induced immune response; usually an increase of target specific cytotoxic T-cells as observed in our trial. Unfortunately, this has not translated into significant survival advantages in phase III trials to date testing antigenic target vaccines, whole cell vaccines and vector based vaccines. In terms of toxicity, all tested vaccines have shown less toxicity compared to immune checkpoint inhibitors and chemotherapies (13–17). The demonstration of enhanced immune response without concomitant survival benefit suggests that vaccine therapy might benefit from combination with other therapeutic modalities such as checkpoint inhibitors, chemotherapy or radiation therapy.

Although the immune checkpoint inhibitors have shown tremendous potential, response rates remain relatively low in lung cancer. Two PD-1 inhibitors (Nivolumab and Pembrolizumab) and one PD-L1 inhibitor (atezolizumab) have been approved by FDA and EMA for 2nd line treatment in NSCLC and Pembrolizumab for first line treatment in patients whose tumors have high expression of PD-L1 (>50%). Durvalumab, a PD-1 inhibitor, is approved by FDA for stage III NSCLC patients post chemoradiotherapy (18). Tumor-associated macrophages (TAM) and MDSCs play important roles in tumor immune evasion and their presence in the tumor limit the accumulation of T-cells. An understanding of IDO-reactive T-cells may lead to a treatment strategy improving effectiveness of checkpoint inhibition by activation of IDO specific T-cells reacting toward both tumor- and regulatory cells at the tumor site, thereby leading to local inflammation and diminished immune inhibition.

We hypothesize that vaccine induced activated IDO-reactive T-cells would attract T-cells into the tumor, resulting in inflammation, inducing PD-L1 upregulation on cancer cells as well as immune cells and thereby generating targets more susceptible to anti-PD-1/PD-L1 immunotherapy.

We therefore suggest that combination of a PD-1 blocking antibody and the IDO derived peptide vaccine potentially could increase clinical benefit in patients with NSCLC. To this end, a clinical phase I/II trial is running at our institution with the combination of an IDO and PD-L1 derived peptide vaccine in combination with Nivolumab for patients with metastatic melanoma. Pre-clinical toxicity data show no additional toxicity with the combination compared to Nivolumab alone (NCT03047928).

Epacadostat an IDO inhibitor plus Pembrolizumab have been tested in patients with NSCLC resulting in response rates up to 40–50% and with no additional toxicities in a phase I/II study (19). Currently a phase III trial (ECHO-305/NCT03322540) is running. However, Epacadostat and Pembrolizumab failed to improve progression free survival compared to Pembrolizumab alone in a phase III trial in patients with metastatic melanoma (ECHO-301/KEYNOTE-252 trial). Extensive biomarker analyses are being conducted to contribute to the understanding of the failure.

Presently, a randomized phase II clinical trial is being initiated in patients with NSCLC combining PD-1 blocking antibody and this IDO derived peptide vaccine (Keynote-764).

## Data availability statement

The datasets generated and/or analyzed during the current study are available from the corresponding author on reasonable request.

## Author contributions

JK performed the experiments, interpreted data, and wrote the paper. IS and MA conceived the project, designed research, interpreted data, and wrote the paper. LE-N, TI, and AM interpreted data and edited the paper.

### Conflict of interest statement

The IDO vaccine is developed by MA. By Danish law on public inventions at public institutions, the Capital Region of Denmark holds the patent, which is licensed for commercialization through the industrial partner IO Biotech. MA and IS are co-founders of IO Biotech. IO Biotech had no role in study design, data collection and analyses or manuscript preparation. The remaining authors declare that the research was conducted in the absence of any commercial or financial relationships that could be construed as a potential conflict of interest.
